# Differential Responses to Blood Pressure and Oxidative Stress in Streptozotocin-Induced Diabetic Wistar-Kyoto Rats and Spontaneously Hypertensive Rats: Effects of Antioxidant (Honey) Treatment

**DOI:** 10.3390/ijms12031888

**Published:** 2011-03-16

**Authors:** Omotayo O. Erejuwa, Siti A. Sulaiman, Mohd Suhaimi Ab Wahab, Kuttulebbai N. S. Sirajudeen, Md Salzihan Md Salleh, Sunil Gurtu

**Affiliations:** 1 Department of Pharmacology, School of Medical Sciences, Universiti Sains Malaysia, 16150 Kubang Kerian, Kelantan, Malaysia; E-Mails: sbsamrah@kb.usm.my (S.A.S.); msuhaimi@kb.usm.my (M.S.A.W.); 2 Department of Chemical Pathology, School of Medical Sciences, Universiti Sains Malaysia, 16150 Kubang Kerian, Kelantan, Malaysia; E-Mail: sirajuden@kb.usm.my; 3 Department of Pathology, School of Medical Sciences, Universiti Sains Malaysia, 16150 Kubang Kerian, Kelantan, Malaysia; E-Mail: matledpb@yahoo.com; 4 Monash University Sunway Campus, Jeffrey Cheah School of Medicine and Health Sciences, Jalan Lagoon Selatan, 46150, Bandar Sunway, Selangor, Malaysia; E-Mail: sgurtu@gmail.com

**Keywords:** hypertension, diabetes mellitus, streptozotocin, spontaneously hypertensive rats, antioxidant enzymes, oxidative stress, kidney, tualang honey

## Abstract

Oxidative stress is implicated in the pathogenesis and/or complications of hypertension and/or diabetes mellitus. A combination of these disorders increases the risk of developing cardiovascular events. This study investigated the effects of streptozotocin (60 mg/kg; ip)-induced diabetes on blood pressure, oxidative stress and effects of honey on these parameters in the kidneys of streptozotocin-induced diabetic Wistar-Kyoto (WKY) and spontaneously hypertensive rats (SHR). Diabetic WKY and SHR were randomized into four groups and received distilled water (0.5 mL) and honey (1.0 g/kg) orally once daily for three weeks. Control SHR had reduced malondialdehyde (MDA) and increased systolic blood pressure (SBP), catalase (CAT) activity, and total antioxidant status (TAS). SBP, activities of glutathione peroxidase (GPx) and glutathione reductase (GR) were elevated while TAS was reduced in diabetic WKY. In contrast, SBP, TAS, activities of GPx and GR were reduced in diabetic SHR. Antioxidant (honey) treatment further reduced SBP in diabetic SHR but not in diabetic WKY. It also increased TAS, GSH, reduced glutathione (GSH)/oxidized glutathione (GSSG) ratio, activities of GPx and GR in diabetic SHR. These data suggest that differences in types, severity, and complications of diseases as well as strains may influence responses to blood pressure and oxidative stress.

## 1. Introduction

Epidemiological studies suggest that hypertension or diabetes mellitus is a major risk factor for cardiovascular disease [1]. It is also known that persons with both hypertension and diabetes mellitus are at a higher risk of developing cardiovascular events than are individuals with either risk factor alone [2]. Similarly, in animal models, chemically induced diabetes produces more profound effects in spontaneously hypertensive rats (SHR) than it does in normotensive strains such as Wistar-Kyoto (WKY) rats [3]. In diabetes mellitus and/or hypertension, the kidney is either a target or it plays a prominent role in these disorders.

The actual mechanisms by which diabetes exacerbates hypertension still remain unclear. However, a number of hypotheses have been postulated to explain some of these mechanisms [4,5]. One of such mechanisms is the role of oxygen-derived free radicals. During atherogenesis, enhanced monocyte adherence to the endothelium, believed to be mediated by induced expression of adhesion molecules and chemotactic proteins, is linked to imbalances or changes in the redox or oxidative state of the endothelial cells [4,6]. In diabetes, hyperglycemia and/or hyperlipidemia is implicated in the increased generation of reactive oxygen species from vascular cells [7,8]. This oxidative stress may trigger a number of oxidant-responsive genes that play a prominent role in monocyte-endothelial cell adhesion resulting in endothelial dysfunction [9–11]. Friedman *et al.* [12] showed that the combination of hypertension and diabetes mellitus is accompanied by higher oxidative stress than that observed in the individual disorder alone [12]. These results would suggest that combined effects of diabetes and hypertension might elicit more pronounced effects not only on redox or antioxidant status but also on their complications compared to the influence of either disease alone.

A number of *ex vivo* studies have documented the antibacterial, antiproliferative, and antioxidant properties of Malaysian tualang honey [13–15]. This honey has been shown by us to produce glucose lowering as well as protective effects against oxidative stress in kidney and pancreas in streptozotocin-induced diabetic Sprague-Dawley rats [16–18]. Since oxidative stress is recognized as a putative factor in the development and complications of both diabetes mellitus and hypertension, it would be interesting to explore the effects of honey in experimental animals with combined diabetic and hypertensive pathologies. In the present study, we have investigated the effects of streptozotocin-induced diabetes on blood pressure, antioxidant enzymes, markers of oxidative stress and the effects of tualang honey on some of these parameters in the kidneys of streptozotocin-induced diabetic WKY and SHR.

## 2. Results and Discussion

### 2.1. Systolic Blood Pressure

**Figure 1.**
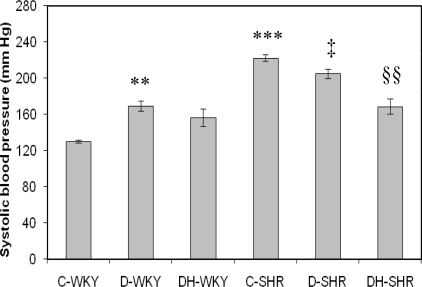
Effect of honey on systolic blood pressure in streptozotocin-induced diabetic WKY and SHR. Each group consisted of five to seven animals. Data are expressed as mean ± SEM. WKY, Wistar-Kyoto rats; SHR, spontaneously hypertensive rats; C-WKY, control WKY; C-SHR, control SHR; D-WKY, diabetic WKY; D-SHR, diabetic SHR; DH-WKY, diabetic WKY + honey; DH-SHR, diabetic SHR + honey. ** p < 0.01, ***p < 0.001 *versus* C-WKY; ‡p < 0.05 *versus* C-SHR; §§p < 0.01 *versus* D-SHR.

Figure 1 shows the systolic blood pressure (BP) in control and STZ-induced diabetic WKY and SHR. Control SHR had significantly (p < 0.001) higher systolic BP than control WKY. Systolic BP was significantly (p < 0.05) reduced in STZ-induced diabetic SHR while it was significantly (p < 0.01) increased in STZ-induced diabetic WKY compared to their corresponding control partners. Treatment with tualang honey further reduced significantly (p < 0.01) BP in STZ-induced diabetic SHR but not in STZ-induced diabetic WKY.

### 2.2. Blood Glucose and Body Weight

**Figure 2.**
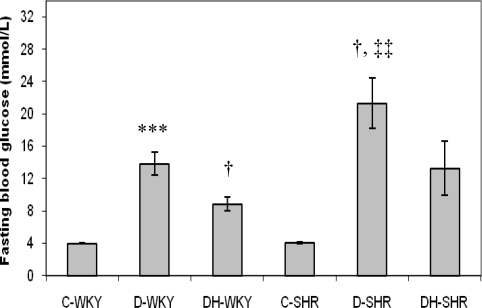
Effect of honey on fasting blood glucose in streptozotocin-induced diabetic WKY and SHR. Each group consisted of five to seven animals. Data are expressed as mean ± SEM. WKY, Wistar-Kyoto rats; SHR, spontaneously hypertensive rats; C-WKY, control WKY; C-SHR, control SHR; D-WKY, diabetic WKY; D-SHR, diabetic SHR; DH-WKY, diabetic WKY + honey; DH-SHR, diabetic SHR + honey. *** p < 0.001 *versus* C-WKY; † p < 0.05 *versus* D-WKY; ‡‡ p < 0.01 *versus* C-SHR.

Figure 2 shows the blood glucose levels in control and STZ-induced diabetic WKY and SHR. Blood glucose concentrations were similar in the control WKY and control SHR. The blood glucose levels were significantly (p < 0.01) elevated in the STZ-induced diabetic WKY and SHR compared to the control WKY and control SHR, respectively. The blood glucose concentrations were significantly (p < 0.05) increased in STZ-induced diabetic SHR compared to STZ-induced diabetic WKY. Treatment with honey significantly (p < 0.05) reduced blood glucose levels in diabetic WKY but not significantly in diabetic SHR.

**Figure 3.**
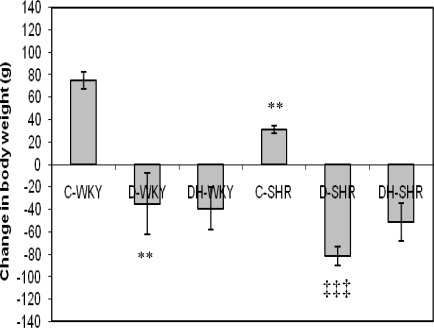
Effect of honey on change in body weight of streptozotocin-induced diabetic WKY and SHR. Each group consisted of five to seven animals. Data are expressed as mean ± SEM. WKY, Wistar-Kyoto rats; SHR, spontaneously hypertensive rats; C-WKY, control WKY; C-SHR, control SHR; D-WKY, diabetic WKY; D-SHR, diabetic SHR; DH-WKY, diabetic WKY + honey; DH-SHR, diabetic SHR + honey. ** p < 0.01 *versus* C-WKY; ‡‡‡ p < 0.001 *versus* C-SHR.

Figure 3 shows change in body weight in control and STZ-induced diabetic WKY and SHR at the end of the treatment period. Before commencement of treatment, SHR had reduced body weight compared to WKY (data not shown). At the end of the treatment period, body weight was significantly (p < 0.01) lower in the control SHR compared to control WKY. With STZ-induced diabetes, body weight was significantly (p < 0.01 and p < 0.001) reduced in WKY and SHR, respectively compared to control rats of the same strain. STZ-induced diabetic SHR lost more weight (but not statistically significant) than STZ-induced diabetic WKY. Treatment of diabetic WKY and SHR with honey produced no effect on body weight.

### 2.3. Antioxidant Enzymes

**Figure 4.**
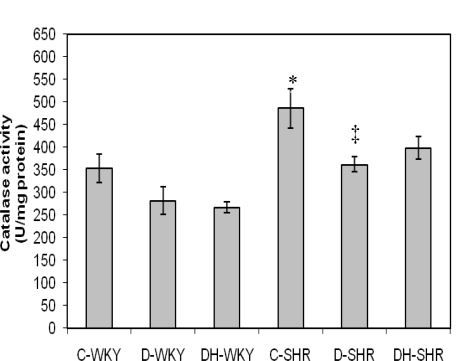
Effect of honey on catalase activity in kidney of streptozotocin-induced diabetic WKY and SHR. Each group consisted of five to seven animals. Data are expressed as mean ± SEM. WKY, Wistar-Kyoto rats; SHR, spontaneously hypertensive rats; C-WKY, control WKY; C-SHR, control SHR; D-WKY, diabetic WKY; D-SHR, diabetic SHR; DH-WKY, diabetic WKY + honey; DH-SHR, diabetic SHR + honey. * p < 0.05 *versus* C-WKY; ‡ p < 0.05 *versus* C-SHR.

**Figure 5.**
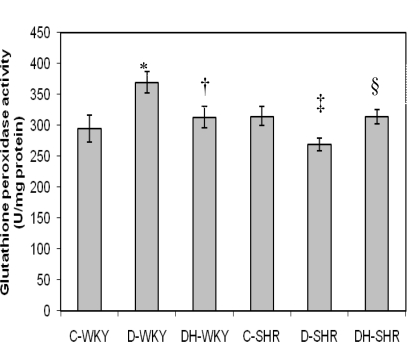
Effect of honey on glutathione peroxidase (GPx) activity in kidney of streptozotocin induced diabetic WKY and SHR. Each group consisted of five to seven animals. Data are expressed as mean ± SEM. WKY, Wistar-Kyoto rats; SHR, spontaneously hypertensive rats; C-WKY, control WKY; C-SHR, control SHR; D-WKY, diabetic WKY; D-SHR, diabetic SHR; DH-WKY, diabetic WKY + honey; DH-SHR, diabetic SHR + honey. * p < 0.05 *versus* C-WKY; † p < 0.05 *versus* D-WKY; ‡ p < 0.05 *versus* C-SHR; §p < 0.05 *versus* D-SHR.

**Figure 6.**
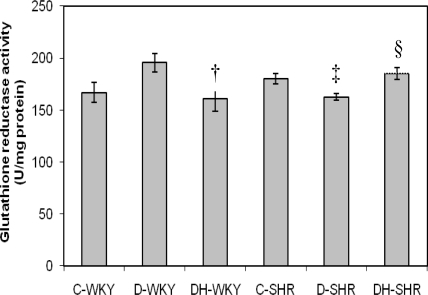
Effect of honey on glutathione reductase (GR) activity in kidney of streptozotocin-induced diabetic WKY and SHR. Each group consisted of five to seven animals. Data are expressed as mean ± SEM. WKY, Wistar-Kyoto rats; SHR, spontaneously hypertensive rats; C-WKY, control WKY; C-SHR, control SHR; D-WKY, diabetic WKY; D-SHR, diabetic SHR; DH-WKY, diabetic WKY + honey; DH-SHR, diabetic SHR + honey. † p < 0.05 *versus* D-WKY; ‡ p < 0.05 *versus* C-SHR; §p < 0.05 *versus* D-SHR.

Figure 4 shows the catalase (CAT) activity in control and STZ-induced diabetic WKY and SHR. Control SHR had significantly (p < 0.05) higher CAT activity compared to control WKY. With STZ-induced diabetes, CAT activity was reduced in both WKY and SHR. The reduced CAT activity was more pronounced in diabetic SHR than in diabetic WKY. Treatment of diabetic WKY and diabetic SHR with honey did not restore CAT activity. Figure 5 shows the GPx activity in control and STZ-induced diabetic WKY and SHR. GPx activity was similar in the control WKY and control SHR. GPx activity was significantly (p < 0.05) elevated in diabetic WKY while it was significantly (p < 0.05) reduced in diabetic SHR. Treatment with honey significantly (p < 0.05) restored GPx activity in both diabetic WKY and diabetic SHR. Figure 6 shows that GR activity was increased though not significantly in diabetic WKY while it was significantly (p < 0.05) reduced in diabetic SHR. Treatment with honey significantly (p < 0.05) reduced GR activity in diabetic WKY while it significantly (p < 0.05) increased GR activity in diabetic SHR. Activities of Superoxide Dismutase (SOD) and Glutathione-S-Transferase (GST) remained the same in the all the groups (data not shown).

### 2.4. Markers of Oxidative Stress

**Figure 7.**
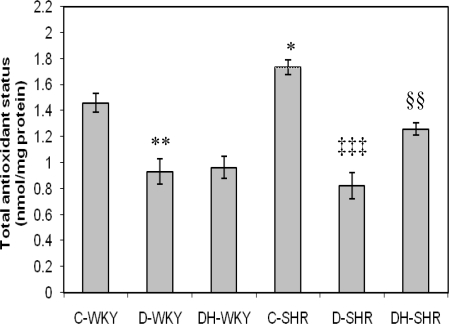
Effect of honey on total antioxidant status (TAS) in kidney of streptozotocin induced diabetic WKY and SHR. Each group consisted of five to seven animals. Data are expressed as mean ± SEM. WKY, Wistar-Kyoto rats; SHR, spontaneously hypertensive rats; C-WKY, control WKY; C-SHR, control SHR; D-WKY, diabetic WKY; D-SHR, diabetic SHR; DH-WKY, diabetic WKY + honey; DH-SHR, diabetic SHR + honey. * p < 0.05, ** p < 0.01 *versus* C-WKY; ‡‡‡ p < 0.001 *versus* C-SHR; §§p < 0.01 *versus* D-SHR.

**Figure 8.**
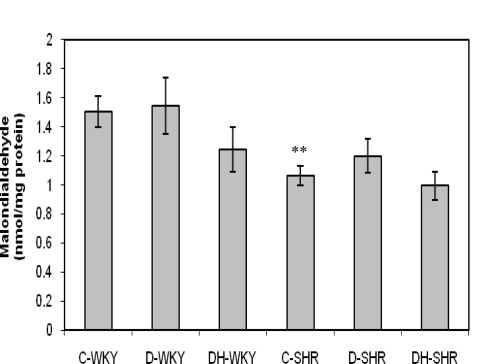
Effect of honey on malondialdehyde (MDA) in kidney of streptozotocin-induced diabetic WKY and SHR. Each group consisted of five to seven animals. Data are expressed as mean ± SEM. WKY, Wistar-Kyoto rats; SHR, spontaneously hypertensive rats; C-WKY, control WKY; C-SHR, control SHR; D-WKY, diabetic WKY; D-SHR, diabetic SHR; DH-WKY, diabetic WKY + honey; DH-SHR, diabetic SHR + honey. ** p < 0.01 *versus* C-WKY.

Figure 7 shows the total antioxidant status (TAS) in control and STZ-induced diabetic WKY and SHR. Control SHR had significantly (p < 0.05) higher TAS than did control WKY. TAS was significantly reduced in diabetic WKY and diabetic SHR (p < 0.01 and p < 0.001, respectively). Treatment with honey significantly (p < 0.01) increased TAS in diabetic SHR but not in diabetic WKY. Figure 8 shows the malondialdehyde (MDA) levels in control and STZ-induced diabetic WKY and SHR. Control SHR had significantly (p < 0.01) lower MDA levels than did control WKY. MDA levels were much lower (though not statistically significant) in the remaining SHR groups compared to corresponding WKY. Treatment with honey further reduced MDA levels but not significantly in both diabetic WKY and diabetic SHR.

**Figure 9.**
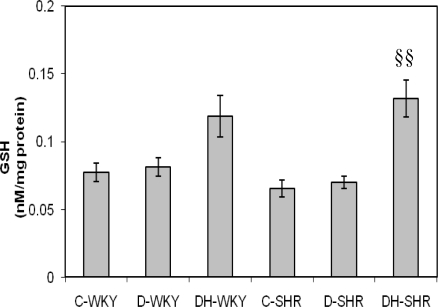
Effect of honey on reduced glutathione (GSH) in kidney of streptozotocin induced diabetic WKY and SHR. Each group consisted of five to seven animals. Data are expressed as mean ± SEM. WKY, Wistar-Kyoto rats; SHR, spontaneously hypertensive rats; C-WKY, control WKY; C-SHR, control SHR; D-WKY, diabetic WKY; D-SHR, diabetic SHR; DH-WKY, diabetic WKY + honey; DH-SHR, diabetic SHR + honey. §§p < 0.01 *versus* D-SHR.

**Figure 10.**
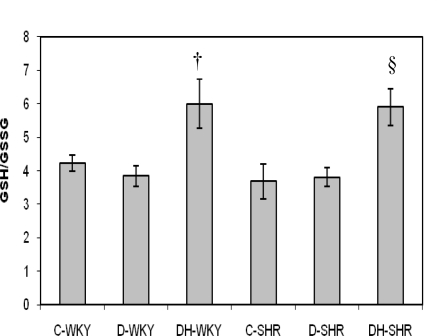
Effect of honey on GSH/GSSG in kidney of streptozotocin induced diabetic WKY and SHR. Each group consisted of five to seven animals. Data are expressed as mean ± SEM. WKY, Wistar-Kyoto rats; SHR, spontaneously hypertensive rats; C-WKY, control WKY; C-SHR, control SHR; D-WKY, diabetic WKY; D-SHR, diabetic SHR; DH-WKY, diabetic WKY + honey; DH-SHR, diabetic SHR + honey. † p < 0.05 *versus* D-WKY; §p < 0.05 *versus* D-SHR.

Figures 9 and 10 show the levels of reduced glutathione (GSH) and GSH/GSSG (oxidized glutathione) ratio in control and STZ-induced diabetic WKY and SHR. The levels of GSH and GSH/GSSG ratio were similar in the control WKY and control SHR. With STZ-induced diabetes, the levels of GSH and GSH/GSSG ratio still remained comparable in the diabetic WKY and diabetic SHR. Treatment with honey increased GSH significantly (p < 0.01) in diabetic SHR but not significantly in diabetic WKY. Also, with honey treatment, GSH/GSSG ratio was increased significantly (p < 0.05) in both diabetic WKY and diabetic SHR. The levels of GSSG remained the same in all the groups (data not shown).

The use of spontaneously hypertensive rats as an animal model to study the pathogenesis and/or complications of hypertension is well documented [19]. Streptozotocin (STZ), an antibiotic derived from *Streptomyces achromogenes*, is a common chemical of choice for inducing animal models of diabetes. Its diabetogenic effect is attributed to its irreversible damage to pancreatic β-cells [20]. The induction of diabetes in normotensive and hypertensive rats serves as an experimental model to investigate the effects of diabetes alone as well as the combined effects of diabetes and hypertension in rats. In this study, STZ-treated Wistar-Kyoto rats (WKY) and spontaneously hypertensive rats (SHR) had significantly increased blood glucose levels compared to control WKY and SHR, respectively as previously reported [21]. In our preliminary results, we had observed that resistance to STZ was higher in WKY than in SHR while mortality was much higher in diabetic SHR than in diabetic WKY [22]. From our data, STZ-induced diabetes had a more profound effect on glycemia in SHR than in WKY. The reason for this is not quite clear. However, some studies have shown that the β-cells are selectively susceptible to STZ, an analog of N-acetylglucosamine (GlcNAc), because they contain higher activity of O-GlcNAc transferase (OGT) (OGT, the enzyme responsible for attaching O-GlcNAc to proteins) more than other cell types [23]. So, it is likely that OGT may be intrinsically over-expressed in SHR β-cells more than in WKY β-cells. There is also a possibility of increased uptake of STZ into SHR β-cells. Moreover, increased oxidative stress is reportedly generated in SHR compared to WKY [24]. Since cytotoxic effect of STZ is believed to be mediated partly through the release of nitric oxide (NO) and reactive oxygen species (ROS) [25,26], administration of STZ might further expose the SHR β-cells to increased levels of free radicals, thus resulting in increased cytotoxicity. These and other mechanisms which require further investigation may play a role in the increased sensitivity of SHR β-cells to STZ. Honey supplementation reduced hyperglycemia significantly in diabetic WKY, similar to what we found in diabetic Sprague-Dawley rats [16–18]. However, honey did not reduce hyperglycemia significantly in diabetic SHR. This might be due to the severity of hyperglycemia or diabetes in these rats. Insulin treatment, in spite of increased serum insulin, also did not reduce hyperglycemia in diabetic SHR [21]. The significantly reduced body weight observed in control SHR is similar to what was reported by other researchers [21]. Our results also showed that in STZ-induced diabetes, the magnitudes of weight loss were more in diabetic SHR than in diabetic WKY. In STZ-induced diabetes, proteolysis, enhanced utilization, and mobilization of lipid in the skeletal muscles are reported to cause weight loss [27,28]. Contrary to our previous findings [16,17], it is quite unclear why honey treatment did not improve body weight in both diabetic WKY and SHR.

Systolic blood pressure (BP) was significantly higher in control SHR than in control WKY similar to what was previously reported [29]. Increased production of superoxide anions is implicated in the pathogenesis of elevated blood pressure in SHR [30]. We also found that STZ-induced diabetic WKY had significantly elevated BP compared to control WKY. Our results are consistent with those of Bunag *et al*. [31]. They found that BP, beginning by the second week, was significantly elevated in STZ-treated rats and the elevated blood pressure persisted through the seventh week. Other researchers also reported similar findings [32]. On the other hand, there are reports of decreased blood pressure [33], or no change [34], with STZ-induced diabetes. The mechanisms by which diabetic normotensive rats become hypertensive or hypotensive still remain poorly understood. At present, it is uncertain whether hypertension is secondary to diabetes or STZ causes hypertension. However, Kawashima *et al*. [35] showed that STZ at different doses, even the lowest dose (20 mg/kg) which did not have an effect on the blood glucose levels, significantly increased blood pressure. Based on their findings, they reported that hyperglycemia is not a prerequisite for induction of hypertension by STZ [35]. In this study, honey treatment in diabetic WKY did not reduce elevated BP. This seems to suggest that the mechanisms of hypertension in STZ-treated WKY might be unrelated to oxidative stress but due to other mechanisms which need further investigation.

With STZ-induced diabetes, BP was significantly reduced in diabetic SHR compared to control SHR. Also, there are conflicting reports on blood pressure in diabetic SHR. Our results are similar to those of Davidoff *et al*. [21], Rodgers [34] and Susic *et al*. [36] who all reported lower blood pressure in diabetic SHR. However, other researchers did report elevated blood pressure [32], or no change in blood pressure [37], in STZ-induced SHR. The hypotensive effect of diabetes in SHR is a consequence of reduced peripheral resistance [36]. The implications of reduced BP in diabetic SHR remain unclear. However, Davidoff and Rodgers [38] reported that decreased blood pressure of diabetic SHR was also accompanied by a progressive decline in heart rate. They further showed that insulin treatment prevented both the depressor and bradycardic effects of diabetes in SHR. Their findings seem to indicate that reduced blood pressure and bradycardia as a result of diabetes in SHR might be detrimental.

Unlike in Davidoff and Rodgers’s study [38], honey supplementation in diabetic SHR resulted in further reduction of BP. Our results corroborate those of Medeiros *et al.* [39] who reported that olive oil or fish oil produced further diminution of blood pressure in diabetic SHR. Olive oil and fish oil, like tualang honey [16–18], are reported to ameliorate oxidative stress [40,41]. In view of the fact that oxidative stress is implicated in the pathogenesis and/or maintenance of hypertension in SHR, the further reduction of BP elicited by these oils or honey might be due to their antioxidant effects. On the other hand, insulin, unknown to exhibit antioxidant effect, might improve diabetes and other metabolic derangements including hyperglycemia. These effects of insulin might diminish or counteract the effects of diabetes on blood pressure resulting in restoration of the BP towards that of the control SHR. Moreover, considering that these researchers reported hyperinsulinemia after a prolonged treatment period, it is possible that elevated insulin levels generated ROS contributing to the steady increments in blood pressure [42].

Our data showed that the activities of GPx and GR were up-regulated in diabetic WKY whereas they were down-regulated in diabetic SHR. Further studies are required to clarify the reasons and also perhaps the implications of these disparities. However, since diabetic WKY compared to diabetic SHR had moderate levels of hyperglycemia, free radicals generated in diabetic WKY might be less compared to those formed in diabetic SHR. As a result, the up-regulated activities of GPx and GR might be a compensatory mechanism to protect the kidneys from the detrimental effects of free radicals [43]. On the other hand, considering the severity of hyperglycemia in diabetic SHR, the kidneys of these rats might be exposed to higher levels of oxidative stress resulting in glycation or inhibition of GPx and GR [44,45]. Besides, elevated blood pressure in SHR might also contribute to increased generation of free radicals [30]. Furthermore, there are reports that differences in sex, tissue, and duration of diseases influence the responses of antioxidant enzymes to changes in redox status [46,47]. Therefore, in diabetic WKY and diabetic SHR, the differential responses of GPx and GR activities (induction or inhibition) to oxidative stress might be attributed to differences in types, severity, and combinations/complications of diseases as well as strains of animals. We found a significantly reduced TAS and a slightly, non-significantly depressed activity of catalase in kidneys of diabetic WKY and diabetic SHR. Other markers of oxidative stress such as MDA, GSH, and GSH/GSSG ratio remained unchanged in kidneys of diabetic WKY and diabetic SHR. The beneficial effects of honey supplementation on some of these markers were noticed in kidneys of diabetic WKY and diabetic SHR.

Our results showed that TAS was significantly higher in control SHR compared to control WKY. Higher TAS is suggestive of lower oxidative stress. In some studies, TAS is reported to be lower in SHR [48]. However, another study found no significant difference in TAS between SHR and WKY [49]. In our study, the reason for the increased TAS in control SHR still remains unclear. Although TAS is supposed to reflect or indicate total antioxidant capacity, this is not always the case. TAS assays generally measure mainly the chain breaking antioxidants such as urate, ascorbate, bilirubin, α-tocopherol, carotenoids, and flavonoids, without taking into consideration the contribution of antioxidant enzymes and other metal binding proteins [50]. Urate, a weak antioxidant *in vivo*, is known to contribute more than 50% of total antioxidant activity in most TAS assays [50]. A study reported that patients suffering from renal failure had increased TAS as a result of elevated urate in spite of the fact that these patients had reduced ascorbate and other chain breaking antioxidants as well as increased free radical damage [51]. Consequently, it is recommended that TAS as an indicator of reduced oxidative stress should be interpreted cautiously because it may mislead and give a false impression of enhanced antioxidant status or defenses [50,51]. Accordingly, we do not believe that higher TAS in control SHR is indicative of reduced oxidative stress. The elevated BP in control SHR also lends credence to this view. The TAS results may corroborate previous findings as well as views expressed by other researchers that TAS, as a marker of oxidative stress, is not sufficient and thus should be coupled with the measurements of antioxidant enzymes, individual antioxidants, and other markers of oxidative damage [50,51].

Whether oxidative stress precedes hypertension or *vice versa* remains controversial. This is because there is evidence in support of either view [30,52]. Our results showed that the control SHR compared to control WKY had significantly increased CAT activity. Considering that SHR, compared to WKY, are known to exhibit increased oxidative stress [12,19,24,30], it is possible that the over-expressed activity of CAT might be a compensatory mechanism to protect the kidney against the deleterious effects of free radicals [43]. We are also of the view that the enhanced CAT activity might contribute considerably to the significantly reduced levels of MDA in control SHR. Surprisingly, the same rats (control SHR) also had significantly elevated BP. Normally, with the reduced levels of MDA, one would have inferred that the levels of oxidative stress were much lower in control SHR than in control WKY. However, it may not be scientifically sound to make such an inference in view of the elevated BP in control SHR coupled with the numerous evidences that support the role of oxidative stress in the development of hypertension or *vice versa* [30,52]. In fact, it is plausible to hypothesize that the reduced levels of MDA and increased TAS in control SHR might be consequences of enhanced CAT activity.

## 3. Materials and Methods

### 3.1. Animals

This study complied with the institutional guidelines for the Care and Use of Animals for Scientific Purposes and was approved by the Animal Ethics Committee, Universiti Sains Malaysia [USM/Animal Ethics Approval/2009/(46) (145)]. Male spontaneously hypertensive rats (SHR) and Wistar-Kyoto (WKY) rats aged 12–14 weeks and weighing 250–300 g were used. The animals were obtained from the Laboratory Animal Research Unit of Universiti Sains Malaysia, Health Campus, Kelantan, Malaysia. The rats were housed in polypropylene cages of one animal per cage. Standard pellet food and water were provided *ad libitum.* The rats were kept in a ventilated animal room with a 12-h dark/12 h-light cycle (lights on 7.00 a.m., lights off 7.00 p.m.) and controlled temperature (25 ± 2 °C).

### 3.2. Chemicals and Tualang Honey

Streptozotocin (STZ), sodium citrate buffer and thiobarbituric acid were obtained from Sigma-Aldrich (St. Louis, MO, U.S.). Superoxide dismutase and glutathione peroxidase assay kits were purchased from Cayman (MI, U.S.). GSH:GSSG assay kit was purchased from Calbiochem (CA, U.S.). Bio-Rad protein assay kit was purchased from Bio-Rad (U.S.). All other chemicals used were of analytical grade obtained from commercial sources.

Tualang honey (AgroMas®, Malaysia) was supplied by Federal Agricultural Marketing Authority (FAMA), Kedah, Malaysia. It has the following composition: total reducing sugar (67.5%) [fructose (29.6%), glucose (30.0%), maltose (7.9%); fructose/glucose ratio (0.99)], sucrose (0.6%) and water (20.0%). Tualang honey was freshly diluted with distilled water just before administration. The daily dose of tualang honey (1.0 g/kg body weight) was chosen based on the findings from our preliminary study [16]. Our subsequent studies have also confirmed this dose to be effective and devoid of any detectable adverse events in pancreas and kidney of diabetic and non-diabetic rats [17,18].

### 3.3. Induction of Diabetes and Treatment

Experimental diabetes was induced in SHR and normotensive WKY rats (250–300 g) by a single intraperitoneal injection of STZ (60 mg/kg body weight; dissolved in sodium citrate buffer, pH 4.5) after an overnight fast (at least 16 hours). Control groups (non-diabetic SHR and WKY rats) received only sodium citrate buffer without STZ. Forty-eight hours after the administration of STZ or citrate buffer, fasting blood glucose concentrations were measured with a portable glucometer (Accu-Chek, Roche, Germany) using a drop of blood from the tail vein. Rats with fasting blood glucose level ≥ 12.0 mmol/L were considered diabetic and included in the study. Diabetic and non-diabetic SHR and WKY rats were randomly assigned to groups of five to seven rats. With the aid of oral gavage, once each morning, the rats were treated with distilled water or tualang honey for three weeks as shown in Table 1.

### 3.4. Measurement of Blood Pressure, Blood Glucose and Body Weight

Systolic blood pressure, blood glucose and body weight were measured before commencement of treatment and at the end of the treatment period. Systolic blood pressure was measured in conscious rats by a tail-cuff plethysmographic non-invasive method (Model 179, Life Science, U.S.). At least four consecutive measurements were recorded and the average was calculated. Fasting blood glucose concentrations were measured using a glucometer (Accu-Chek, Roche, Germany). The body weight was measured with the aid of a weighing balance. After treatment for 3 weeks, the animals were fasted for at least 16 hours and sacrificed by decapitation.

### 3.5. Processing of Tissues and Biochemical Assays

The left kidney was rapidly excised, washed in ice-cold normal saline, blotted and quickly stored at −80 °C until use. 10% (w/v) homogenate of kidney was prepared in Tris-HCl (0.1 M, pH 7.4) using an ice-chilled glass homogenizing vessel in a homogenizer fitted with Teflon pestle (Glas-Col, U.S.) at 900 rpm. The suspended mixture was centrifuged at 1000 × *g* for 10 min at 4 °C in a refrigerated centrifuge. The resulting supernatant was used for the assay of total protein, activities of antioxidant enzymes and other markers of oxidative stress.

### 3.6. Superoxide Dismutase (SOD) Assay

SOD activity was determined using Cayman assay kit according to manufacturer’s instruction. This assay kit utilizes a tetrazolium salt for the detection of superoxide radicals generated by xanthine oxidase and hypoxanthine. One unit of SOD is defined as the amount of enzyme needed to exhibit 50% dismutation of superoxide radical. The SOD assay measures all the three types of SOD (Cu/Zn, Mn, and FeSOD).

### 3.7. Catalase (CAT) Assay

CAT activity was measured according to the method of Goth [53]. Briefly, this assay involves the incubation of sample test tube containing 0.5 mL of hydrogen peroxide and 0.1 mL of kidney homogenate. After incubation at 37 °C for 60 seconds, the enzymatic reaction was stopped by addition of 0.5 mL of a Mmonium molybdate solution. The yellow complex of ammonium molybdate and hydrogen peroxide was then measured spectrophotometrically at 405 nm. One unit of CAT was defined as the amount of enzyme that catalyzes the decomposition of 1 μmol of hydrogen peroxide per minute.

### 3.8. Glutathione Peroxidase (GPx) Assay

GPx activity was measured using Cayman assay kits according to manufacturer’s instructions. This kit measures GPx activity indirectly by a coupled reaction with glutathione reductase (GR). Oxidized glutathione (GSSG), produced upon reduction of hydroperoxide by GPx, is recycled to its reduced state by GR and NADPH. The oxidation of NADPH is accompanied by a decrease in absorbance at 340 nm. One unit of GPx is defined as the amount of enzyme that catalyzes the oxidation of 1 nmol of NADPH per minute at 25 °C.

### 3.9. Glutathione-S-Transferase (GST) Assay

GST activity was assayed according to the method of Habig *et al* [54]. Briefly, 2 mL of 0.3 M potassium phosphate buffer (pH 6.35), 75 μL of 30 mM CDNB solution, 725 μL of distilled water and 0.1 mL of kidney homogenate were pipetted into a test tube. The test tube was vortexed and incubated at 37 °C for 10 minutes. After incubation, the reaction was initiated by addition of 100 μL of 30 mM reduced glutathione solution. The decrease in absorbance was measured spectrophotometrically at 340 nm and recorded every 30 seconds for 4 minutes. One unit of GST was defined as the amount of enzyme that catalyzes the conjugation of 1 nmol of GSH-CDNB per minute.

### 3.10. Glutathione Reductase (GR) Assay

GR activity was assayed according to the method of Goldberg and Spooner [55]. Briefly, 1 mL of 2.728 mM GSSG solution and 40 μL of kidney homogenate were incubated for 5 minutes at 37 °C. After incubation, the reaction was initiated by addition of 200 μL of 1.054 mM NADPH solution. The decrease in absorbance was measured at 340 nm using a spectrophotometer (Unico UV-2800, China) and recorded every 30 seconds over a period of 5 minutes. One unit of GR was defined as the amount of enzyme that catalyzes the oxidation of 1 nmol of NADPH per minute. One unit of GR was defined as the amount of enzyme that catalyzes the oxidation of 1 nmol of NADPH per minute.

### 3.11. Total Antioxidant Status (TAS) Assay

TAS was measured according to the method of Koracevic *et al.* [56]. 10 μL of kidney homogenate was pipetted in a test tube containing 0.49 mL of 100 mM sodium phosphate buffer. This was followed by the addition of 0.5 mL of 10 mM sodium benzoate solution, 0.2 mL of Fe-EDTA mixture and 0.2 mL of 10 mM H_2_O_2_ solution. Negative control (with phosphate buffer instead of the kidney homogenate) containing similar reagents as in sample test tubes was also prepared. The test tubes were vortexed and incubated at 37 °C for 60 minutes. This was followed by the addition of 1 mL of 20% acetic acid and 0.8% (w/v) thiobarbituric acid (TBA). The reaction tubes were incubated at 100 °C for 10 minutes. After cooling to room temperature, the absorbance of the mixture was measured spectrophotometrically at 532 nm against distilled water. TAS in the kidney homogenates was calculated using uric acid as standard. TAS was expressed as nmol uric acid equivalent per mg protein.

### 3.12. Lipid Peroxidation Assay

Lipid peroxidation was determined as malondialdehyde (MDA) accordin*g* to the method of Ohkawa *et al*. [57]. For this, 100 μL of kidney homogenates or MDA standards were pipetted into test tubes containing 1.5 mL of 20% (w/v) glacial acetic acid (pH 3.5), 200 μL of 8.1% (w/v) sodium dodecyl sulphate (SDS), 1.5 mL of 0.8% (w/v) TBA and 700 μL of distilled water. After incubation at 95 °C for 60 minutes, the test tubes were cooled and then centrifuged at 3000 × g for 10 minutes. The MDA concentration was measured at 532 nm using a spectrophotometer (Unico UV-2800, China). 1,1,3,3-Tetraethoxypropane (TEP) was used as MDA standard. The MDA concentration was expressed as nmol MDA per mg protein.

### 3.13. Assay of Reduced and Oxidized Glutathione

Total glutathione and oxidized glutathione (GSSG) were estimated using GSH:GSSG ratio assay kit (Calbiochem, CA, U.S.) according to the manufacturer's instructions. Reduced glutathione (GSH) and GSH/GSSG ratio were then calculated. Briefly, the kidney homogenates were deproteinized in 5% metaphosphoric acid, centrifuged and the supernatants were used for the estimation of glutathione. For GSSG assay, the kidney homogenates were immediately mixed with the thiol-scavenging reagent, 1-methy-2-vinylpyridinium trifluoromethanesulfonate to derivatize GSH. Samples were mixed sequentially with the chromogen 5,5′-dithiobis-2-nitrobenzoic acid, glutathione reductase and NADPH. The absorbance of each sample was recorded at 412 nm and the reaction rate was determined. A standard curve was constructed using a known quantity of GSH standards. GSH and GSSG concentrations were calculated by linear regression against the standard curve. GSH/GSSG ratio was calculated using the formula [ratio = (GSH − 2GSSG)/GSSG]. GSH and GSSG concentrations were expressed as nmol of glutathione per mg protein.

### 3.14. Protein Assay

Protein concentration was estimated using Bio-Rad protein assay kit based on the method of Bradford [58]. The assay is a dye-binding assay in which a differential color change of a dye, with maximum absorbance at 595 nm, occurs in response to various concentrations of protein. A standard curve was obtained using bovine serum albumin.

### 3.15. Statistical Analysis

Statistical analysis was carried out using SPSS 18.0.1. Data were analyzed using independent t test to identify significance of difference between two groups. Data are expressed as mean ± SEM. P value < 0.05 was considered to be statistically significant.

## 4. Conclusions

If hyperglycemia is not a prerequisite for the development of high blood pressure in diabetic normotensive rats [35], then oxidative stress in diabetic normotensive rats may be a consequence of hypertension and/or diabetes. Furthermore, our data seem to suggest that oxidative stress, unlike in SHR, may not contribute considerably to elevated blood pressure in STZ-treated diabetic rats. Though additional studies with other potent antioxidants such as tempol are required, the inability of tualang honey to decrease elevated BP of diabetic WKY further substantiates this view. The effectiveness of antioxidants including tualang honey not only in ameliorating oxidative stress but also in reducing elevated BP in diabetic SHR corroborate the prominent role of ROS in elevated BP of SHR. Our results indicate that differences in types, severity, and combinations/complications of diseases (hypertension and/or diabetes), as well as strains, may influence antioxidant enzymes in response to changes in redox status. Our study underscores the need for additional studies to elucidate the mechanisms by which STZ-induced diabetes causes hypertension in diabetic normotensive rats. Further studies that investigate the protein and gene expressions of GPx and GR may reveal a differential role in regulating post-translational modification of these antioxidant enzymes in diabetic WKY and diabetic SHR.

## Figures and Tables

**Table 1.** Treatment groups.

**Group Treatment**
Non-diabetic WKY Distilled water (0.5 mL)
Diabetic WKY Distilled water (0.5 mL)
Diabetic WKY Tualang honey (1.0 g/kg/body weight)
Non-diabetic SHR Distilled water (0.5 mL)
Diabetic SHR Distilled water (0.5 mL)
Diabetic SHR Tualang honey (1.0 g/kg/body weight)

## References

[1] Kannel WB, McGee DL (1978). Diabetes and cardiovascular disease: The Framingham study. JAMA.

[2] Christlieb AR (1973). Diabetes and hypertensive vascular disease. Am. J. Cardiol.

[3] Fein FS, Capasso JM, Aronson RS, Cho S, Nordin C, Miller-Green B, Sonnenblick EH, Factor SM (1984). Combined renovascular hypertension and diabetes in rats: A new preparation of congestive cardiomyopathy. Circulation.

[4] Ross R (1986). The pathogenesis of atherosclerosis. N. Engl. J. Med.

[5] Reaven GM (1995). Pathophysiology of insulin resistance in human disease. Physiol. Rev.

[6] Pieper GM, Gross GJ (1988). Oxygen-derived free radicals abolish endothelium-dependent relaxation in diabetic rat aorta. Am. J. Physiol.

[7] Tesfamariam B, Cohen RA (1992). Free radicals mediate endothelial cell dysfunction caused by elevated glucose. Am. J. Physiol.

[8] Ohara Y, Petersen TE, Harrison DG (1993). Hypercholesterolemia increases endothelial O_2_^−^ anion production. J. Clin. Invest.

[9] Marui N, Offerman MK, Swerlick R, Kunsch C, Rosen CA, Ahmad M, Alexander RW, Medford RM (1993). Vascular cell adhesion molecule-1 (VCAM-1) gene transcription and expression are regulated through an antioxidant-sensitive mechanism in human vascular endothelial cells. J. Clin. Invest.

[10] Laight DW, Desai KM, Anggard EE, Carrier MJ (2000). Endothelial dysfunction accompanies a pro-oxidant, pro-diabetic challenge in the insulin resistant, obese Zucker rat *in vivo*. Eur. J. Pharmacol.

[11] Weber C, Erl W, Pietsch A, Strobel M, Ziegler-Heitbrock HWL, Weber PC (1994). Antioxidants inhibit monocyte adhesion by suppressing nuclear factor-κB mobilization and induction of vascular cell adhesion molecule-1 in endothelial cells stimulated to generate radicals. Arterioscler. Thromb.

[12] Friedman J, Peleg E, Kagan T, Shnizer S, Rosenthal T (2003). Oxidative stress in hypertensive, diabetic, and diabetic hypertensive rats. Am. J. Hypertens.

[13] Tan HT, Rahman RA, Gan SH, Halim AS, Hassan SA, Sulaiman SA, Kirnpal-Kaur B (2009). The antibacterial properties of Malaysian tualang honey against wound and enteric microorganisms in comparison to manuka honey. BMC Complement. Altern. Med.

[14] Ghashm AA, Othman NH, Khattak MN, Ismail NM, Saini R (2010). Antiproliferative effect of tualang honey on oral squamous cell carcinoma and osteosarcoma cell lines. BMC Complement. Altern. Med.

[15] Mohamed M, Sirajudeen KNS, Swamy M, Yaacob NS, Sulaiman SA (2010). Studies on the antioxidant properties of tualang honey of Malaysia. Afr. J. Trad. CAM.

[16] Erejuwa OO, Gurtu S, Sulaiman SA, Ab Wahab MS, Sirajudeen KNS, Salleh MS (2010). Hypoglycemic and antioxidant effects of honey supplementation in streptozotocin-induced diabetic rats. Int. J. Vitam. Nutr. Res.

[17] Erejuwa OO, Sulaiman SA, Ab Wahab MS, Sirajudeen KNS, Salzihan MS, Gurtu S (2010). Antioxidant protective effect of glibenclamide and metformin in combination with honey in pancreas of streptozotocin-induced diabetic rats. Int. J. Mol. Sci.

[18] Erejuwa OO, Sulaiman SA, Ab Wahab MS, Sirajudeen KNS, Salzihan MS, Gurtu S (2011). Comparison of antioxidant effects of honey, glibenclamide, metformin, and their combinations in kidney of streptozotocin-induced diabetic rats. Int. J. Mol. Sci.

[19] Trippodo NC, Frolich ED (1981). The genetically hypertensive rat as a model for human essential hypertension. Circ. Res.

[20] Rakieten N, Rakieten ML, Nadkarni MV (1963). Studies on the diabetogenic action of streptozotocin (NSC-37917). Cancer Chemother. Rep.

[21] Davidoff AJ, Pinault FM, Rodgers RL (1990). Ventricular relaxation of diabetic spontaneously hypertensive rats. Hypertension.

[22] Erejuwa OO, Sulaiman SA, Ab Wahab MS, Sirajudeen KNS, Salzihan MS, Gurtu S (2010).

[23] Roos MD, Wen X, Kaihong S, Clark JA, Xiaoyong Y, Chin E, Paterson AJ, Kudlow JE (1998). Streptozotocin, an analog of N-acetylglucosamine, blocks the removal of O-GlcNAc from intracellular proteins. Proc. Assoc. Am. Phys.

[24] Suzuki H, Swei A, Zweifach BW, Schmid-Schonbein GW (1995). *In vivo* evidence for microvascular oxidative stress in spontaneously hypertensive rats: Hydroethidine microfluorography. Hypertension.

[25] Haluzik M, Nedvidkova J (2000). The role of nitric oxide in the development of streptozotocin-induced diabetes mellitus: experimental and clinical implication. Physiol. Res.

[26] Takasu N, Komiya I, Asawa T, Nagasawa Y, Yamada T (1991). Streptozotocin- and alloxan-induced H_2_O_2_ generation and DNA fragmentation in pancreatic islets. H_2_O_2_ as mediator for DNA fragmentation. Diabetes.

[27] Pamela CC, Richard AH (1994). Biochemistry.

[28] Stearns SB, Tepperman HM, Tepperman J (1979). Studies on the utilization and mobilization of lipid in skeletal muscles from streptozotocin-diabetic and control rats. J. Lipid Res.

[29] Factor SM, Bhan R, Minase T, Wolinsky H, Sonnenblick EH (1981). Hypertensive-diabetic cardiomyopathy in the rat. An experimental model of human disease. Am. J. Pathol.

[30] Touyz RM, Schiffrin EL, Holtzman J (2008). Oxidative stress in arterial hypertension: oxidative stress and hypertension. Atherosclerosis and Oxidant Stress—A New Perspective.

[31] Bunag RD, Tomita T, Sasaki S (1982). Streptozotocin diabetic rats are hypertensive despite reduced hypothalamic responsiveness. Hypertension.

[32] Sharma JN, Kesavarao U (1996). Cardiac kallikrein in hypertensive and normotensive rats with and without diabetes. Immunopharmacology.

[33] To mLinson KC, Gardiner SM, Bennett T (1990). Blood pressure in streptozotocin-treated Brattleboro and Long Evans rats. Am. J. Physiol.

[34] Rodgers RL (1986). Depressor effect of diabetes in the spontaneously hypertensive rat: Associated changes in heart performance. Can. J. Physiol. Pharmacol.

[35] Kawashima H, Igarashi T, Nakajima Y, Akiyama Y, Usuki K, Ohtake S (1978). Chronic hypertension induced by streptozotocin in rats. Naunyn-Schmiedebergs Arch. Pharmacol.

[36] Susic D, Mandal AK, Jovovic DJ, Radujkovic G, Kentera D (1990). Streptozotocin-induced diabetes mellitus lowers blood pressure in spontaneously hypertensive rat. Clin. Exp. Hypertens. A.

[37] Kelly DJ, Wilkinson-Berka JL, Allen TJ, Cooper ME, Skinner SL (1998). A new model of diabetic nephropathy with progressive renal impairment in the transgenic (mRen-2)27 rat (TGR). Kidney Int.

[38] Davidoff AJ, Rodgers RL (1990). Insulin, thyroid hormone, and heart function of diabetic spontaneously hypertensive rat. Hypertension.

[39] Medeiros FJ, Aguila MB, Mandarim-de-Lacerda CA (2006). Renal cortex remodeling in streptozotocin-induced diabetic spontaneously hypertensive rats treated with olive oil, palm oil and fish oil from Menhaden. Prostaglandins Leukot. Essent. Fatty Acids.

[40] Moreno JJ, Carbonell T, Sánchez T, Miret S, Mitjavila MT (2001). Olive oil decreases both oxidative stress and the production of arachidonic acid metabolites by the prostaglandin G/H synthase pathway in rat macrophages1. J. Nutr.

[41] Nyby MD, Abedi K, Eslami P, Hernandez G, Smutko V, Berger ME, Tuck ML (2004). Dietary fish oil prevents hypertension, oxidative stress and suppression of endothelial nitric oxide synthase expression in fructose-fed rats. Am. J. Hypertens.

[42] Ge X, Yu Q, Qi W, Shi X, Zhai Q (2008). Chronic insulin treatment causes insulin resistance in 3T3-L1 adipocytes through oxidative stress. Free Radic. Res.

[43] Tiedge M, Lortz S, Munday R, Lenzen S (1999). Protection against the co-operative toxicity of nitric oxide and oxygen free radicals by over-expression of antioxidant enzymes in bioengineered insulin-producing RI nm5F cells. Diabetologia.

[44] Wolff SP, Dean RT (1987). Glucose autoxidation and protein modification: The potential role of autoxidative glycosylation in diabetes. Biochem. J.

[45] Pigeolet E, Corbisier P, Houbion A, Lambert D, Michiels C, Raes M, Zachary MD, Remacle J (1990). Glutathione peroxidase, superoxide dismutase and catalase inactivation by peroxides and oxygen derived free radicals. Mech. Ageing Dev.

[46] Sartori-Valinotti JC, Iliescu R, Fortepiani L, Yanes L, Reckelhoff J (2007). Sex differences in oxidative stress and the impact on blood pressure control and cardiovascular disease. Clin. Exp. Pharm. Physiol.

[47] Maritim AC, Sanders RA, Watkins JB (2003). Diabetes, oxidative stress and antioxidants: A review. J. Biochem. Mol. Toxicol.

[48] Newaz MA, Nawal NN (1998). Effect of alpha-tocopherol on lipid peroxidation and total antioxidant status in spontaneously hypertensive rats. Am. J. Hypertens.

[49] Mantle D, Patel VB, Why HJ, Ahmed S, Rahman I, MacNee W, Wassif WS, Richardson PJ, Preedy VR (2000). Effects of lisinopril and a mLodipine on antioxidant status in experimental hypertension. Clin. Chim. Acta.

[50] Young IS (2001). Measurement of total antioxidant capacity. J. Clin. Pathol.

[51] Jackson P, Loughrey CM, Lightbody JH, McNamee PT, Young IS (1995). Effect of hemodialysis on total antioxidant capacity and serum antioxidants in patients with chronic renal failure. Clin. Chem.

[52] Nabha L, Garbern JC, Buller CL, Charpie JR (2005). Vascular oxidative stress precedes high blood pressure in spontaneously hypertensive rats. Clin. Exp. Hypertens.

[53] Gott L (1991). A simple method for determination of serum catalase activity and revision of reference range. Clin. Chim. Acta.

[54] Habig WH, Pabst MJ, Jakoby WB (1974). Glutathione-S-transferases. The first enzymatic step in mercapturic acid formation. J. Biol. Chem.

[55] Goldberg DM, Spooner RJ, Bergmeyen HV (1983). Assay of glutathione reductase. Methods of Enzymatic Analysis.

[56] Koracevic D, Koracevic G, Djordjevic V, Andrejevic S, Cosic V (2000). Method for the measurement of antioxidant activity in human fluids. J. Clin. Pathol.

[57] Ohkawa H, Ohishi N, Yagi K (1979). Assay for lipid peroxides in animal tissues by thiobarbituric acid reaction. Anal. Biochem.

[58] Bradford MA (1976). Rapid and sensitive method for the quantitation of microgram quantities of protein utilizing the principle of protein-dye binding. Anal. Biochem.

